# Enhanced MAPK signaling drives ETS1-mediated induction of miR-29b leading to downregulation of TET1 and changes in epigenetic modifications in a subset of lung SCC

**DOI:** 10.1038/onc.2015.499

**Published:** 2016-01-18

**Authors:** M A Taylor, M Wappett, O Delpuech, H Brown, C M Chresta

**Affiliations:** 1 AstraZeneca, R&D Oncology iMed, Alderley Park, Macclesfield, UK; 2 Personalised Healthcare and Biomarkers, AstraZeneca, Alderley Park, Macclesfield, UK

## Abstract

Non-small-cell lung cancer is the leading cause of cancer death worldwide and is comprised of several histological subtypes, the two most common being adenocarcinoma (AC) and squamous cell carcinoma (SCC). Targeted therapies have successfully improved response rates in patients with AC tumors. However, the majority of SCC tumors lack specific targetable mutations, making development of new treatment paradigms for this disease challenging. In the present study, we used iterative non-negative matrix factorization, an unbiased clustering method, on mRNA expression data from the cancer genome atlas (TCGA) and a panel of 24 SCC cell lines to classify three disease segments within SCC. Analysis of gene set enrichment and drug sensitivity identified an immune-evasion subtype that showed increased sensitivity to nuclear factor-κB and mitogen-activated protein kinase (MAPK) inhibition, a replication-stress associated subtype that showed increased sensitivity to ataxia telangiectasia inhibition, and a neuroendocrine-associated subtype that showed increased sensitivity to phosphoinositide 3-kinase and fibroblast growth factor receptor inhibition. Additionally, each of these subtypes exhibited a unique microRNA expression profile. Focusing on the immune-evasion subtype, bioinformatic analysis of microRNA promoters revealed enrichment for binding sites for the MAPK-driven ETS1 transcription factor. Indeed, we found that knockdown of ETS1 led to upregulation of eight microRNAs and downregulation of miR-29b in the immune-evasion subtype. Mechanistically, we found that miR-29b targets the DNA-demethylating enzyme, TET1, for downregulation resulting in decreased 5-hmC epigenetic modifications. Moreover, inhibition of MAPK signaling by gefitinib led to decreased ETS1 and miR-29b expression with a corresponding increase in TET1 expression and increase in 5-hmC. Collectively, our work identifies three subtypes of lung SCC that differ in drug sensitivity and shows a novel mechanism of miR-29b regulation by MAPK-driven ETS1 expression which leads to downstream changes in TET1-mediated epigenetic modifications.

## Introduction

Non-small-cell lung cancer is the most prevalent type of lung cancer in the world and the leading cause of cancer death, accounting for 1.4 million deaths annually.^1^ Non-small-cell lung cancer consists of several histological subtypes, the two most common being adenocarcinoma (AC) and squamous cell carcinoma (SCC).^2^ While targeted therapies, such as those that target epidermal growth factor receptor (EGFR), have been successful in improving response rates in patients with AC tumors, the majority of SCC tumors lack specific targetable mutations. One challenge in defining treatment paradigms for SCC is the high level of heterogeneity within this disease. Gene expression profiling has improved our understanding of cancer and led to the development of multigene signatures that predict outcomes and response to therapy.^3, 4, 5^ However, such signature classifications have not changed treatment for SCC. Therefore, the development of therapies targeted for SCC will depend on gaining a greater understanding of the molecular underpinnings that drive tumorigenesis and progression in this disease setting.

MicroRNAs are small (20–30 nucleotide) non-coding RNAs that can function as either tumor promoters or suppressors during tumorigenesis by exerting post-transcriptional effects on gene expression.^6^ Additionally, microRNAs are often expressed in a tissue- and disease-specific manner,^7^ making them ideal candidates as biomarkers.^7, 8^ In this study, we used global gene expression profiling to define subtypes present within lung SCC. Importantly, we found each of these subtypes to have a unique therapeutic sensitivity and microRNA expression profile. We demonstrate that the ETS1 transcription factor, driven by pathways enriched in the immune-evasion subtype, drives the differential expression of a subset of microRNAs expressed in this subtype. Through this analysis, we identified miR-29b as a microRNA whose expression is driven by ETS1, through activated mitogen-activated protein kinase (MAPK) signaling. Additionally, we found that miR-29b targets the 5-hydroxymethylcytosine dioxygenase, TET1 for downregulation and has downstream effects on TET1-mediated epigenetic modifications.

## Results

### Iterative non-negative matrix factorization clustering of lung SCC reveals three genomic subtypes with unique drug sensitivity and cell signaling profiles

**Figure 1:**
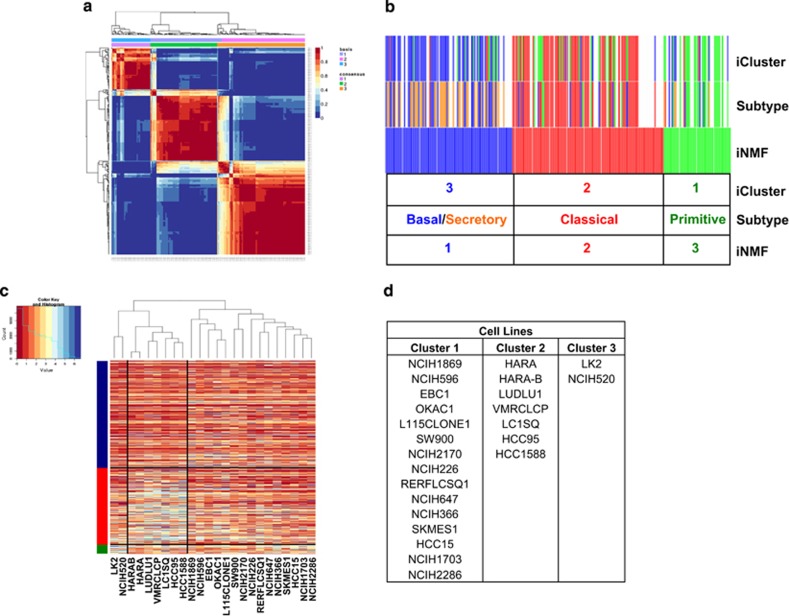
iNMF clustering to identify subtypes of lung SCC. (**a**) iNMF was used to uncover three core subtypes of gene expression differences within 258 samples of lung SCC obtained from The Cancer Genome Atlas (TCGA). (**b**) The map showing overlap of individual TCGA samples clustered by iCluster,^12^ Subtype^13^ and iNMF. Cluster 1 is indicated in blue, cluster 2 in red and cluster 3 in green. (**c**, **d**) iNMF clusters were applied to a panel of 24 SCC cell lines.

In order to classify genomically distinct subtypes within lung SCC, we employed iterative non-negative matrix factorization (iNMF), an unbiased clustering technique, chosen for its ability to overcome the limitations of consensus clustering and provide higher resolution than hierarchical clustering.^9, 10^ iNMF was applied to mRNA expression data from 258 SCC patient samples available from the cancer genome atlas (TCGA), revealing three subtypes, driven by a specific subset of genes (Supplementary Table S1 and Figure 1a). This technique was validated using an independent data set,^11^ which also resulted in three subtypes, with similar gene enrichment profiles (Supplementary Figure S1 and Supplementary Table S2). Despite the differences in gene expression between the subtypes, there was no statistical difference in survival proportion, tumor stage or smoking status between these subtypes (Supplementary Figure S2). Similar to our work, iNMF iClustering performed by TCGA research network yielded three consensus clusters.^12^ Additionally, a previous study, using the consensus plus clustering method reported four subtypes of SCC designated as classical, basal, secretory and primitive.^13^ Comparison of our analysis to these studies indicated a high degree of overlap between the three methods (Figure 1b and Supplementary Figure S3), with cluster 1 most closely aligning with the previously reported basal and secretory subtypes, cluster 2 with the classical subtype and cluster 3 with the primitive subtype. Differences in clustering likely reflect differences in patient sample numbers, patient population and differences in methodology. Having demonstrated that our methods agreed with previously published methods performed on different data sets, we applied hierarchical clustering based on the features identified by iNMF to a panel of 24 lung SCC cell lines (GSE57083) to model these clinically relevant subtypes in cell line models of disease (Figure 1c). Fifteen cell lines closely aligned with patient samples from cluster 1, seven cell lines aligned with cluster 2 and two cell lines aligned with cluster 3 (Figure 1d).

Next, we investigated whether cell lines belonging to each subtype differed in sensitivity to targeted therapies. To do this, we devised a compound sensitivity score where the average negative log GI50 of a compound across all cell lines was subtracted from the average negative log GI50 of cell lines in each cluster. A negative value was indicative that cell lines in a particular cluster were more resistant to a compound than the panel as a whole, while a positive value indicated that cluster cell lines were more sensitive to a compound than the panel as a whole. We found that each cluster exhibited a unique drug response profile (Table 1 and Supplementary Table S3).

To better understand the underlying molecular pathways driving the differential drug sensitivity between subtypes, we used gene set enrichment analysis (GSEA) to determine the signaling pathways most active in each cluster in both TCGA and cell line samples (Table 2 and Supplementary Table S4). Cluster 1 samples were enriched for EGFR, MAPK and nuclear factor-κB signaling. Nuclear factor-κB signaling is known to subvert the immune system within the tumor micro-environment.^14^ Moreover, enhanced signaling through this pathway due to EGFR and KRAS mutations has been shown to upregulate programmed death-ligand 1 contributing to tumor immune escape.^15, 16, 17, 18^ Indeed, both cluster 1 TCGA and cell line samples that had high levels of signaling through EGFR/MAPK pathways showed higher levels of programmed death-ligand 1 (Supplementary Figure S4). Based on this evidence we named cluster 1 the immune-evasion subtype. Interestingly, cell lines from this immune-evasion subtype, which had increased EGFR and nuclear factor-κB signaling also showed enhanced sensitivity to inhibition of EGFR/MAPK signaling pathways by gefitinib (EGFR tyrosine-kinase inhibitor), AZD9291 (EGFR tyrosine-kinase inhibitor) and the MEK 1/2 inhibitor selumetinib (AZD6244, ARRY-142886). In addition, this cell line cluster showed enhanced sensitivity to nuclear factor-κB inhibition by AZD2230 (Table 1).

GSEA of cluster 2 indicated that these patient samples and cell lines were enriched for genes regulated by the transcription factor NRF2. Additionally, GSEA identified high expression of genes upregulated by knockdown of eIF4G1, a translation initiation factor linked to nutrient sensing by mammalian target of rapamycin (mTOR),^19^ suggesting decreased mTOR signaling in cluster 2 (Table 2). Concordantly, cell lines from this subtype showed resistance to mTOR inhibition by AZD2014 (Table 1). Another key feature of cluster 2 samples was enrichment for DNA synthesis and replicative stress (Table 2). Tumors harboring enhanced levels of replication stress are dependent on ataxia telangiectasia-mediated replication responses for their survival.^20^ Indeed, cluster 2 cell lines showed enhanced sensitivity to ataxia telangiectasia inhibition by AZD6738 (Table 1). Given this evidence, we entitled this cluster the replication-stress-associated subtype.

Cluster 3 showed enrichment for signatures associated with a de-differentiated state: stem cell, β-catenin and neuronal signatures (Table 2). Similar to the neuroendocrine subtype of small cell lung cancers, these tumors also displayed upregulation of the neuroendocrine markers chromogranin A and chromogranin B (Supplementary Figure S4), leading us to name this cluster the neuroendocrine subtype. Both cell lines in this neuroendocrine subtype are known to have copy number gains in fibroblast growth factor receptor 1 indicating enhanced fibroblast growth factor receptor 1 signaling and explaining sensitivity to fibroblast growth factor receptor inhibition by AZD4547 in these cells. Additionally, these neuroendocrine cell lines showed sensitivity to AKT, phosphoinositide 3-kinase and mTOR inhibition with AZD5363, GDC0941 and AZD2014 compounds, respectively (Table 1). Neuroendocrine tumors from small cell lung and colorectal cancers also display an enhanced sensitivity to mTOR pathway inhibition, indicating that the phenomenon may apply more broadly to other neuroendocrine cancers.^21, 22, 23^

### SCC genomic subtypes have distinct microRNA expression profiles

**Figure 2:**
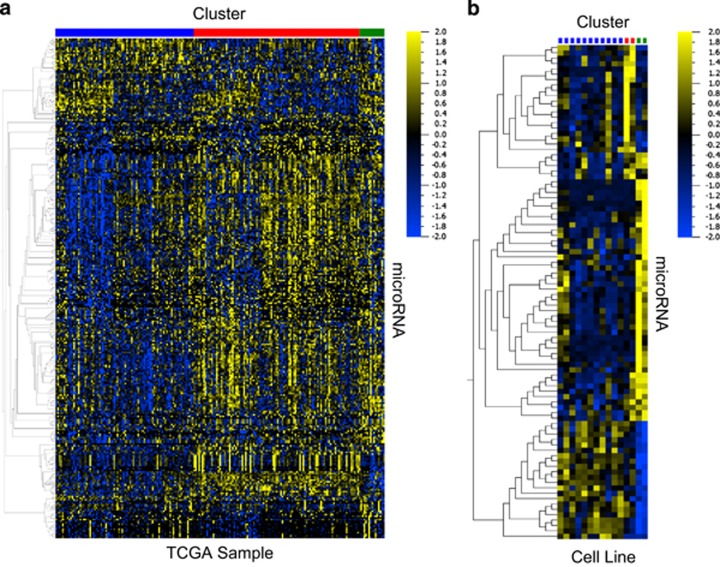
SCC genomic subtypes have distinct microRNA expression profiles. (**a**) The most differentially expressed microRNAs between the three iNMF clusters in TCGA (ANOVA, *P*<0.05). (**b**) The most differentially expressed microRNAs in the SCC cell lines (ANOVA, *P*<0.05). Cluster 1 (immune-evasion) is indicated in blue, cluster 2 (replication-stress) is indicated in red and cluster 3 (neuroendocrine) is indicated in green.

To determine if differences in cell signaling in each subtype drive differences in microRNA expression, we used analysis of variance (ANOVA) analysis with a cutoff of *P*<0.05 to define the microRNAs that are most differentially expressed between all three subtypes in the TCGA data set (Figure 2a) and found 306 differentially expressed microRNAs (Supplementary Table S5). Next, total RNA was extracted from a subset of the cell line panel (15 cell lines) and subjected to microRNA profiling. ANOVA analysis was performed using a cutoff of *P*<0.05 and we found 92 microRNAs differentially expressed between the subtypes in our representative cell lines (Figure 2b and Supplementary Table S5). By combining these two lists, we determined which microRNAs most clearly defined each subtype in both the TCGA samples and cell lines (Table 3). Overall, these data show that there are molecularly distinct subtypes present in SCC that have distinct differences in microRNA expression, which may be useful for identifying subtypes.

### Transcription factors predicted to modulate microRNA expression are differentially expressed across the three subtypes

**Figure 3:**
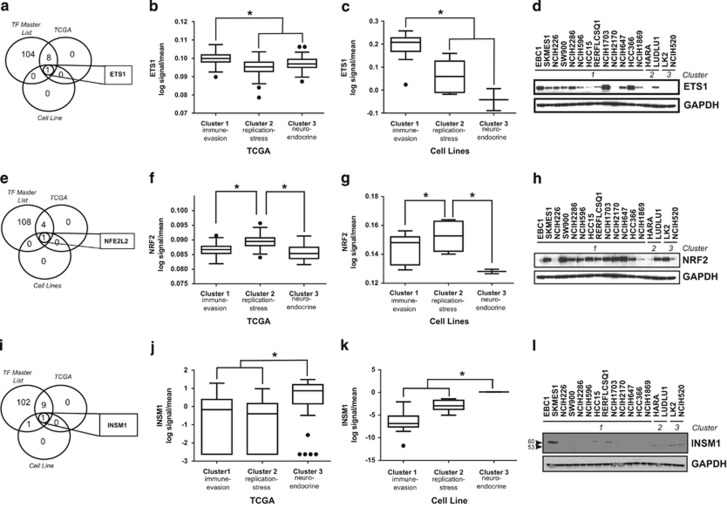
Transcription factors predicted to modulate microRNA expression are differentially expressed across the three subtypes. (**a**) Venn diagram depicting the overlap of transcription factors predicted to bind the promoters of microRNAs differentially regulated between subtypes (TF master list), transcription factors significantly upregulated in cluster 1 TCGA samples and transcription factors significantly upregulated in cluster 1 cell lines compared with the other two clusters. (**b**) Expression of ETS1 in TCGA samples grouped by subtype. (**c**) Expression of ETS1 in cell lines grouped by subtype. (**d**) Immunoblotting of detergent-solubilized whole-cell extracts with ETS1 antibody or GAPDH antibody. (**e**) Venn diagram depicting the overlap of the TF master list, transcription factors significantly upregulated in cluster 2 TCGA samples, and transcription factors significantly upregulated in cluster 2 cell lines compared with the other two clusters. (**f**) Expression of NRF2 in TCGA samples grouped by subtype. (**g**) Expression of NRF2 in cell lines grouped by subtype. (**h**) Immunoblotting of detergent-solubilized whole-cell extracts with NRF2 antibody or GAPDH antibody. (**i**) Venn diagram depicting the overlap of the TF master list, transcription factors significantly upregulated in cluster 3 TCGA samples and transcription factors significantly upregulated in cluster 3 cell lines compared with the other two clusters. (**j**) Expression of INSM1 in TCGA samples grouped by subtype. (**k**) Expression of INSM1 in cell lines grouped by subtype. (**l**) Immunoblotting of detergent-solubilized whole-cell extracts with INSM1 antibody or GAPDH antibody. *P<0.05, Student's *t*-test and all western blots are a representative image from three independent experiments.

MicroRNA gene sequences are located in various genomic contexts, such as introns, non-coding or coding transcripts, and exonic regions. While much work has been done in recent years to characterize microRNA-target regulation, how the majority of microRNAs are regulated at the level of transcription is only beginning to be elucidated. To better understand how the enriched signaling pathways might be driving differences in microRNA expression, we interrogated the predicted promoter regions of microRNAs differentially expressed between the three clusters and compiled a ‘master list' of transcription factors predicted to regulate these microRNAs (Supplementary Table S6). We then examined the expression of these transcription factors in both TCGA data and cell lines. In the immune-evasion subtype ETS1 was the only predicted transcription factor that was significantly upregulated compared with the other subtypes (Figure 3a) at the level of mRNA expression in both TCGA (Figure 3b) and cell line (Figure 3c) data. Moreover, ETS1 protein expression was higher in cell lines belonging to the immune-evasion subtype (Figure 3d). ETS1 expression has previously been shown to be driven by EGFR and MAPK signaling,^24, 25^ suggesting that the enhanced EGFR/MAPK signaling in the immune-evasion subtype drives expression of ETS1, which may then drive differences in microRNA expression in this subtype. Consistent with the replication-stress-associated subtype having an enrichment for genes regulated by NRF2 (Table 2), NFE2L2 (NRF2) was the only predicted transcription factor significantly upregulated compared with the other two subtypes (Figure 3e) at the level of mRNA expression in both TCGA (Figure 3f) and cell line (Figure 3g) data. However, protein expression of NRF2 was relatively high in cell lines from all three subtypes (Figure 3h). In the neuroendocrine subtype, the transcription factor INSM1, known to be highly expressed in tumors of neuroendocrine orgin,^26^ was the only predicted transcription factor significantly upregulated compared with both of the other two subtypes (Figure 3i) at the level of mRNA in both TCGA (Figure 3j) and cell line (Figure 3k) data. Additionally, protein expression of INSM1 (53kda) was high in cell lines belonging to this subtype (Figure 3l).

### The transcription factor ETS1 modulates expression of microRNAs in the immune-evasion subtype

**Figure 4:**
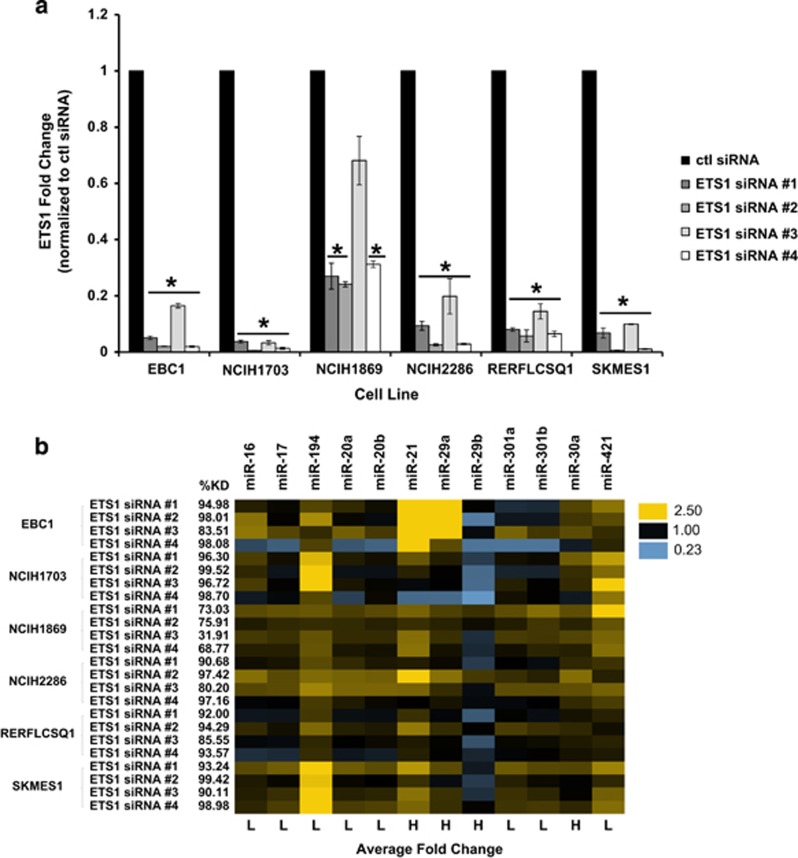
ETS1 modulates expression of microRNAs in the immune-evasion subtype. (**a**) Control (non-targeting) or ETS1 specific (ETS1 siRNA #1, #2, #3 and #4) siRNAs were transfected into indicated cell lines and after 72 h, decreased expression of ETS1 was confirmed by semiquantitative real-time PCR (data are the mean±s.e.m. of *n*=3, *P<0.05, Student's *t*-test). (**b**) Fold change in microRNA expression after ETS1 knockdown compared with control siRNA was measured by fluidigm chip PCR (*n*=3). microRNAs with low expression in the original signature are indicated with an ‘L', while those with high expression are indicated in an ‘H' under the heatmap. Percent knockdown under each experimental condition is indicated to the left of the heatmap. Only microRNAs that were significantly (*P*<0.05) changed in at least one cell line are shown (see Supplementary Table S7 for complete statistical results).

To test the supposition that ETS1, NRF2 and INSM1 were modulating the differential expression of microRNAs in each subtype, we depleted each of these transcription factors using siRNA, then measured changes in microRNA expression in cell lines from each subtype. Depletion of NRF2 and INSM1did not result in consistent microRNA changes between cell lines (data not shown), suggesting that additional co-factors or transcription factors contribute to regulation of microRNA expression in these clusters. For this reason, we focused our attention on microRNA changes induced by ETS1 in immune-evasion subtype cell lines. We depleted ETS1, using four separate siRNAs which resulted in a significant decrease in transcript levels across six cell lines from the immune-evasion subtype (Figure 4a). Next, we measured fold change in microRNA expression resulting from this ETS1 depletion. In general, there was a trend towards microRNAs that had low expression in the original signature being upregulated (Figure 4b). Indeed, ETS1 depletion resulted in significant upregulation of miR-16, miR-17, miR-194, miR-20a, miR-20b, miR-301a, miR-301b and miR-421, suggesting that ETS1 represses expression of these microRNAs (Figure 4b and Supplementary Table S7). Of the four microRNAs that were shown to be highly expressed in the immune-evasion subtype as compared with the other subtypes (miR-21, miR-30a, miR-29a and miR-29b), miR-29b was significantly decreased by ETS1 depletion (Figure 4b and Supplementary Table S7), suggesting that ETS1 drives the expression of miR-29b.

### MiR-29b targets TET1 for downregulation

**Figure 5:**
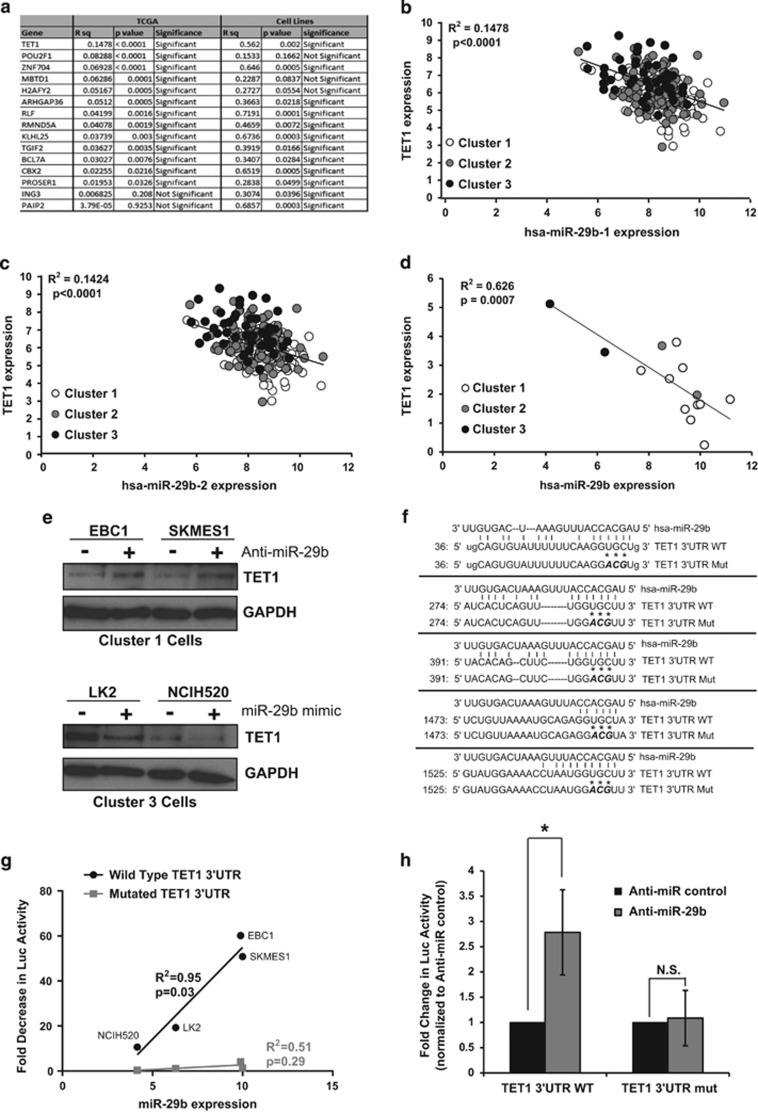
miR-29b targets TET1 for downregulation. (**a**) Regression analysis of 15 mRNA targets of miR-29b identified by Ingenuity Pathway Analysis. *R*^2^ and *P*-values for inverse correlation between miR-29b and each gene are shown for both TCGA samples and cell lines (significant=*P*<0.05). (**b**) Regression plot of TET1 mRNA expression compared with hsa-miR-29b-1 expression in TCGA samples indicates significant inverse correlation *P*<0.0001. (**c**) Regression plot of TET1 mRNA expression compared with hsa-miR-29-2 expression in TCGA samples indicates significant inverse correlation *P*<0.0001. (**d**) Regression plot of TET1 mRNA expression compared with hsa-miR-29b expression in cell line samples indicates a significant degree of inverse correlation *P*=0.0007. (**e**) Immunoblotting of detergent-solubilized whole-cell extracts with TET1 antibody 48 h after transfection with either Anti-miR-29b (or non-targeting Anti-miR control) or miR-29b mimic (or control mimic). Differences in protein loading were monitored by reprobing stripped membranes with antibody against GAPDH. Shown is a representative image from three independent experiments. (**f**) Alignment of has-miR-29b to the wild type and mutant TET1 3′UTR sequences. Asterisks indicate mutated miR-29b seed sequence-binding bases. (**g**) Cells were transiently transfected with a luciferase reporter construct containing no 3′UTR, wild-type TET1 3′UTR or a mutated TET1 3′UTR sequence. Luciferase signal was normalized to the construct with no 3′UTR and the fold decrease in signal was plotted against the miR-29b expression levels of each cell line indicating a significant (*P*=0.03) correlation between miR-29b expression and decrease in wild-type TET1 luciferase reporter, but not the mutated reporter (*P*=0.29) (*n*=3). (**h**) EBC1 cells were transiently co-transfected with Anti-miR-29b (or Anti-miR control) and either a wild-type TET1 3′UTR reporter or a mutated TET1 3′UTR reporter (TET1 3′UTR mut) and luciferase signal was normalized to Anti-miR control transfected cells. Data are the mean±s.e.m. of *n*=3, *P<0.05, Student's *t*-test.

Given the inverse expression of miR-29b observed between the immune-evasion and the neuroendocrine subtypes (Table 3) and the modulation in expression of this microRNA by ETS1 (Figure 4b), we chose this microRNA for further investigation. To identify mRNA targets of miR-29b, we used Ingenuity Pathway Analysis software to compare predicted targets of miR-29b that showed decreased expression in the immune-evasion subtype, where miR-29b levels are high, and increased expression in the neuroendocrine subtype where miR-29b levels are low in both the TCGA and cell line data sets. This identified 15 possible mRNA targets of miR-29b (Figure 5a). Of these, the 5-methylcytosine dioxygenease TET1 enzyme that facilitates DNA demethylation by converting 5-methylcytosine (5mC) bases to 5-hydroxymethylcytosine (5-hmC)^27^ was the most significantly inversely correlated to miR-29b (Figure 5a). MiR-29b is transcribed from two genomic loci (miR-29b-1 and miR-29b-2), both of which showed inverse correlation with TET1 expression in TCGA tumors (Figures 5b and c). Additionally, TET1 expression was inversely correlated with miR-29b expression in SCC cell lines (Figures 5a and d). Transfection of an Anti-miR-29b into immune-evasion subtype cell lines, EBC1 and SKMES1, where miR-29b levels are high, led to an increase in TET1 protein levels (Figure 5e). Conversely, transfection of an miR-29b mimic into neuroendocrine subtype cells LK2 and NCIH520, where miR-29b levels are low led to decreased expression of TET1 protein (Figure 5e). Examination of the TET1 3′untranslated region (UTR) revealed that it contains five miR-29b binding sites (Figure 5f). To determine if miR-29b-mediated downregulation of TET1 occurs through miR-29b binding to the TET1 3′UTR, we measured luciferase reporter activity using constructs containing either a wild-type TET1 3′UTR or a mutated TET1 3′UTR (Figure 5f) and compared activity with a construct with no 3′UTR. The high basal levels of miR-29b expression in immune-evasion subtype cell lines EBC1 and SKMES1 directly correlated with greater decreases in TET1 3′UTR-luciferase reporter activity compared with neuroendocrine subtype cells LK2 and NCIH520, which contain low levels of miR-29b (Figure 5g). This correlation was not observed when the miR-29b binding sites in the TET1 3′UTR-luciferase reporter were mutated (Figures 5f and g). Additionally, co-transfection of Anti-miR-29b with the TET1 3′UTR-luciferase reporter in EBC1 cells led to increased expression of luciferase containing the TET1 3′UTR. Importantly, mutating the miR-29b seed sequence-binding region of the TET1 3′UTR abrogated this increase in luciferase expression (Figure 5h). Taken together, these results indicate that miR-29b targets TET1 for downregulation through an interaction with the TET1 3′UTR.

### miR-29b-mediated downregulation of TET1 leads to changes in the epigenetic modification 5-hmC

**Figure 6:**
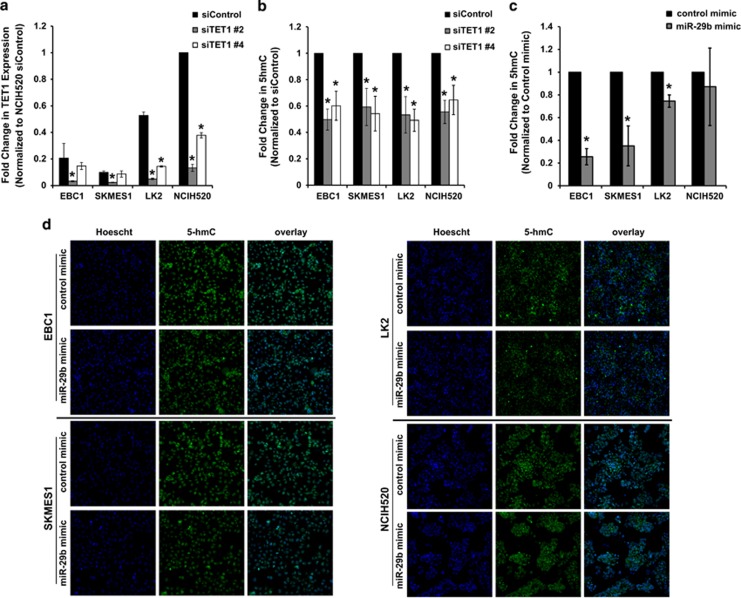
miR-29b-mediated downregulation of TET1 leads to changes in the epigenetic modification 5-hmC. (**a**) Cells were transiently transfected with siRNA against TET1 or control siRNA and knockdown was determined by semi-quantitative real-time PCR, where individual signals were normalized to 18S. (**b**) After transfection with siRNA against TET1 or control siRNA cells were immunostained for 5-hmC and Hoescht and an algorithm measuring nuclear fluorescence intensity of 5-hmC was used to quantitate the percent of cells with 5-hmC in the nucleus and plotted as fold change to siRNA control (**c**) Cells were transiently transfected with miR-29b mimic or control mimic, after 48 h cells were immunostained for 5-hmC and Hoescht and quantitated as in (**b**). (**d**) Representative images of immunostaining after transfections with control mimic or miR-29b mimic. All experiments are the mean±s.e.m. *n*=3, *P<0.05, Student's *t*-test.

TET1 is highly expressed in embryonic stem cells where it plays an important role in pluripotency, self renewal and differentiation^28^ by preventing both aberrant methylation spreading and stochastic hypermethylation.^29, 30^ Given the high expression of TET1 and the stem-like features in the neuroendocrine subtype compared with the other two subtypes, we investigated if there were differences in DNA methylation between subtypes. While we did not observe any difference in genome-wide methylation between subtypes (data not shown), we observed lower levels of DNA methylation in the genes identified by iNMF in the neuroendocrine subtype compared with the other two subtypes (Supplementary Figure S5 and Supplementary Table S8), suggesting that high levels of TET1 in this subtype may contribute to decreased methylation of genes driving the subtypes. To examine how changes in TET1 expression might contribute to gene expression signatures driving the subtypes, we compared our iNMF gene signature (Supplementary Table S1) with TET1 knockdown signatures from previously published studies.^29, 30, 31^ We then compiled a list of 39 genes that showed differential expression between the immune-evasion subtype and the neuroendocrine cell lines and that had been previously reported to be modulated by TET1 depletion (Supplementary Figure S6). Knockdown of TET1 in LK2 and NCIH520 cells where TET1 expression is high (Figure 6a) resulted in a trend towards increased expression of a subset of the genes that were low, and a decrease in a subset of genes that were high after 72 h. Although we did observe a very slight increase in expression of EGFR, this small increase was unable to reprogram and resensitize these resistant cell lines to gefitinib (Supplementary Figure S7 and Supplementary Table S9). Since TET1 is known to modulate DNA demethylation patterns by converting 5mC into 5-hmC,^27^ we confirmed that knockdown of TET1 (Figure 6a) led to decreased 5-hmC (Figure 6b). Next, we tested if miR-29b expression altered 5-hmC levels within the nucleus of cells. In line with miR-29b leading to downregulation of TET1, overexpression of miR-29b led to significantly decreased 5-hmC levels in the nucleus of EBC1, SKMES1 and LK2 cells (Figures 6c and d).

### MAPK signaling regulates miR-29b-mediated downregulation of TET1and leads to changes in 5-hmC levels

**Figure 7:**
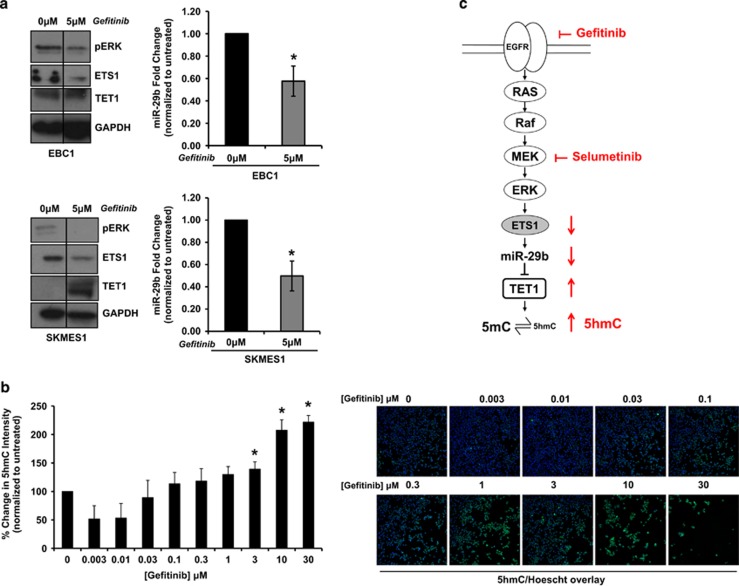
MAPK signaling regulates miR-29b-mediated downregulation of TET1and leads to changes in 5-hmC levels. (**a**) Cell lines were treated with 5 μm gefitinib or DMSO for 72 h. Afterwards, miR-29b levels were monitored by semiquantitative real-time PCR (±s.e.m., *n*=3) and immunoblotting was performed to measure differences in phospho-ERK, ETS1 and TET1. Differences in protein loading were monitored by reprobing stripped membranes with antibody against GAPDH. Shown is a representative image from three independent experiments. (**b**) Cells were treated with increasing concentrations of gefitinib for 48 h after which point, cells were immunostained for 5-hmC and Hoescht and visualized using a x10 objective on the cellomics Cell Insight. An algorithm measuring the nuclear fluorescence intensity of 5-hmC was used to analyze the cells. Data are the mean±s.e.m. of *n*=3,*P<0.05, Student's *t*-test. (**c**) High levels of EGFR/MAPK signaling in the immune-evasion subtype leads to an upregulation of ETS1 which drives increased expression of miR-29b. miR-29b targets TET1 for downregulation through interacting with its 3′UTR, which leads to decreased 5-hmC levels indicative of increased DNA methylation.

Given the well-established role of ETS1 as an effector of MAPK signaling^24, 25^ and our evidence that ETS1 upregulates miR-29b expression (Figure 4b), we hypothesized that EGFR/MAPK signaling in the immune-evasion subtype would drive miR-29b expression through ETS1 leading to suppression of TET1. To test this hypothesis, we treated EBC1 and SKMES1 immune-evasion subtype cell lines with the EGFR inhibitor gefitinib, which led to a reduction in MAPK signaling, indicated by decreased phosphorylation of ERK1/2 (Figure 7a). Concomitant with this decrease in MAPK signaling, we observed a decrease in ETS1 and miR-29b and a corresponding increase in TET1 levels (Figure 7a). Additionally, gefitinib treatment of EBC1 cells also led to increased 5-hmC levels in the nucleus (Figure 7b). Interestingly, gefitinib treatment of EBC1 and SKMES1 also resulted in gene expression changes in the opposite direction to TET1 knockdown for a subset of genes (Supplementary Figure S6 and Supplementary Table S10). Collectively, these results indicate ETS1 and miR-29b expression are driven by MAPK signaling and that abrogation of miR-29b-mediated suppression of TET1 by MAPK pathway inhibition leads to increased levels of 5-hmC and may impact downstream methylation and gene expression.

## Discussion

Here, we demonstrate that lung SCC is composed of three subtypes that are driven by diverse cell signaling pathways, exhibit differential microRNA expression and differential drug sensitivity profiles. Additionally, we found that the immune-evasion subtype is enriched for EGFR signaling, which drives the expression of the transcription factor ETS1. In turn, ETS1 drives the upregulation of miR-29b, leading to the downregulation of TET1 and downstream decreases in 5-hmC epigenetic modifications (Figure 7c).

Targeted therapies against EGFR have been one of the most successful therapeutic strategies in lung AC patients with activating mutations in EGFR.^2^ In contrast, lung SCC generally lack activating mutations in *EGFR*^32^ and while a small fraction of SCC patients, without activating *EGFR* mutations, do respond to EGFR targeted therapies, a predictive biomarker for response to EGFR targeted therapies in SCC has yet to be identified.^33^ Interestingly, we have found that while very few immune-evasion subtype patients and cell lines have mutations in EGFR, there is an overall enrichment for signaling through the EGFR and MAPK signaling pathways. Additionally, We have uncovered a novel role for the MAPK-driven transcription factor ETS1 in regulating the expression of miR-29b, which suggests that miR-29b could be a biomarker for ERK/MAPK pathway activation.

It is well established that epigenetic changes can lead to promotion of tumor initiation and progression.^34^ The role of DNA methyltransferases, which promote DNA methylation, in driving cancer progression have been well characterized.^34^ However, until the recent discovery of the TET family of proteins, the role of DNA demethylation in cancer was less well characterized. The TET family is thought to act as tumor suppressors by maintaining other tumor suppressor genes in their unmethylated state.^31, 35^ Indeed, loss of function mutations in TET2 are frequently found in hematological malignancies and have been implicated in promoting tumor progression.^35^ Recently, analysis of microRNA-target mRNA expression correlations across 11 human cancer types in TCGA showed that TET1 was significantly inversely correlated with miR-29b across all cancer types.^36^ We provide mechanistic evidence that miR-29b directly downregulates TET1 expression (Figure 5). At present, there are ~80 validated targets of miR-29b^37, 38^ with one of the most well characterized being the DNA methyltransferases DNMT3A and DNMT3B.^39^ Taken together this suggests that miR-29b may act to fine tune DNA methylation status by protecting against aberrant changes in both methylating and demethylating enzymes.

Previous work has shown that suppression of TET1 expression is essential for KRAS-induced DNA hypermethylation and that this occurs through MAPK signaling.^31^ We provide further evidence that TET1 is downregulated through MAPK signaling and that this occurs through the novel mechanism of ETS1-mediated upregulation of miR-29b, which targets TET1 for downregulation through an interaction with its 3′UTR (Figures 4 and 5), leading to downstream effects on 5-hmC levels (Figures 6 and 7). Several studies have shown synergy between histone deacetylase inhibition and MAPK inhibition^40, 41^ as well as synergy between DNA-hypomethylating agents like 5-azacytidine and MAPK inhibitors,^42, 43^ suggesting that DNA demethylation enhances the efficacy of MAPK inhibitors. More recently TET upregulation has been shown to be essential for active demethylation induced by 5-azacytidine.^44^ This suggests that drug treatments that upregulate TET1 may synergize with MAPK suppression. However, given our finding that neuroendocrine subtype cell lines are not sensitive to EGFR/MAPK inhibition, high TET1 expression in the absence of MAPK signaling pathway activation is not likely to indicate sensitivity to MAPK inhibition.

## Materials and methods

### Cell culture and constructs

Cell lines were grown in RPMI 1640 media+10% fetal calf serum+2 mm glutamine at 37 °C 5% carbon dioxide and authenticated at AstraZeneca cell banking using DNA fingerprinting short-tandem repeat assays. MiRIDIAN miR-29b microRNA mimic (50 nm final concentration) or hairpin inhibitor (100 nm final concentration) were obtained from Thermo Scientific (Waltham, MA, USA) and ON-TARGETplus siRNA constructs were obtained from Dharmacon (Lafayette, CO, USA). All were transiently transfected using RNAiMAX (Invitrogen, Carlsbad, CA, USA) according to the manufacturer's recommendations.

### iNMF

RNA-seq data in RSEM format from the 258 sample TCGA lung SCC gene expression data set^12^ where read data were collapsed down to the gene level was analyzed in R using the package NMF.^45^ Prior to NMF, genes that were expressed at log2<0 across all samples were removed. Factorization rank estimation was performed, estimating the approximate matrix for up to a maximum of 10 metagenes (or clusters) using 50 runs and benchmarked against randomized data to prevent over fitting. With a cophenetic correlation score of ~0.94, the three cluster solution was selected for the full NMF run using the brunet NMF algorithm and 200 runs. The genes and samples associated with each of the metagenes were extracted. The associated genes were used to separate the cell line data (GSE57083) into clusters using the heatmap.2 function from the R package gplots. For the validation data set,^11^ only SCC sample data were extracted for analysis, data were RMA normalized and NMF was performed on the probeset level and mapped to genes following analysis.

### microRNA expression

Total RNA was purified from cell lines using the miRNeasy kit (Qiagen, Valencia, CA, USA), and 100 ng was labeled and hybridized to the array using the miRNA complete labeling and hyb kit (Agilent, Santa Clara, CA, USA) and spike-in kit (Agilent). Data were generated on a GeneChip Scanner 3000 (Agilent), Human microRNA Microarray Release 16.0, 8x60K arrays (Agilent). Data were imported into R, RMA normalized using the package AgiMicroRna,^46^ summarized to the log2 scale and returned for further association analysis as a gene by sample matrix. The miR expression data are MIAME compliant and have been submitted to the Gene Expression Omnibus (GSE73774). Differences in microRNA expression were determined with Omics Explorer (Qlucore, Lund, Sweden), using ANOVA on 549 miRs from TCGA^12^ and 1368 miRs from cell lines using using a cutoff of *P*<0.05 and false discovery rate of 0.09 and 0.73, respectively, calculated using the Benjamini–Hochberg method. Fold change of miRs found to be significant (*P*<0.05) in both TCGA and cell lines was then determined by comparing expression in each cluster with the other two clusters (Table 3).

### GSEA and transcription factor predictions

High-throughput mRNA sequencing data from 258 lung SCC cancer patients were downloaded from the TCGA data portal. GSEA was performed using software (v2.1.0) obtained from the Broad Institute using the c2 and c6 databases from the MsigDB. *P*-values, enrichment scores and *q-*values were computed by permuting the sample labels (cluster number) 1000 times.^47, 48^ The ACTViewer program (Qu lab at Sun Yat-sen University) was used to identify transcription factor-binding sites in the promoter regions of differentially expressed microRNAs.

### Real-time PCR

Total RNA was purified using the supplementary microRNA protocol for the RNeasy Plus Mini Kit (Qiagen). Expression of mRNA targets was measured using QuantiTect one-step reverse transcriptase-PCR reagents (Qiagen) and primer/probe sets from Life Technologies on a LightCycler 480 (Roche, Basel, Switzerland). For microRNA, cDNAs were synthesized using miScript II Reverse Transcription Kit (Qiagen) and diluted fivefold in RNase-free water prior to preamplification using the miScript Microfluidics PreAMP kit (Qiagen) and oligonucleotide primers from Life Technologies. Real-time PCR was carried out using reagents from the Microfluidics qPCR kit (Qiagen) on a Fluidigm 48 × 48 chip or carried out from 10-fold diluted cDNA following the miScript PCR System protocol (Qiagen) on a LightCycler480 (Roche). Primers and probes are listed in Supplementary Table S11.

### Luciferase assays

Cells were seeded into 96-well plates and co-transfected using TransIT-X2 (Mirus, Madison, WI, USA) with microRNA mimic (Thermo Scientific) or Anti-miR (Thermo Scientific) along with 50 ng of pMirTarget (Origene, Rockville, MD, USA) housing the 3′UTR sequence of TET1 that contained either a wild-type or mutant version of the miR-29b binding site. Luciferase and RFP expression were measured using the Steady Glo Luciferase kit (Promega, Madison, WI, USA) and a Tecan microplate reader.

### *In vitro* assays

Western blotting was performed as previously described.^49^ Antibodies are listed in Supplementary Table S12. Proliferation and dose response assays were performed by plating cells into a 96-well plate. Confluence was measured every 4 h using the IncuCyte (Essen Bioscience, Ann Arbor, MI, USA) for 6 days and data were analyzed in Graph Pad Prism 6.

### Immunofluorescence

Cells were seeded into a clear bottom, black wall 96-well plate (then transfected with microRNA mimic (Thermo Scientific) Anti-miR (Thermo Scientific), or 20 nm siRNA (Dharmacon) using RNAiMAX (Invitrogen) or treated with drug. Cells were fixed with 3.7% formaldehyde, permeabilized in 0.5% Triton-X-100, denatured with 2 n HCl and neutralized with 100 mm Tris-HCl (pH8.5), then blocked with 5% BSA/0.1% Triton X-100 before probing with an antibody against 5-hydroxymethylcytosine (5-hmC) (Active Motif, Carlsbad, CA, USA) overnight at 4 °C. Following washing in phosphate-buffered saline-Tween 0.05%, cells were incubated with a secondary antibody conjugated to Alexa-Fluor-488 (Invitrogen) and Hoescht (Invitrogen) before washing and imaging using a × 10 objective on the Cellomics Cell Insight. An algorithm measuring the nuclear fluorescence intensity was used for analysis.

### Methylation analysis

Methylation by mean *β-*values were downloaded from TCGA and Omics Explorer (Qlucore) was used to perform ANOVA to determine genes that were differentially methylated.

### Statistics

Statistical values were defined using ANOVA or a two-tailed Student's *t*-test as specified in the text, *P*<0.05 was considered significant and distribution was assumed to be normal for all *in vitro* experiments.

## Figures and Tables

**Table 1:**
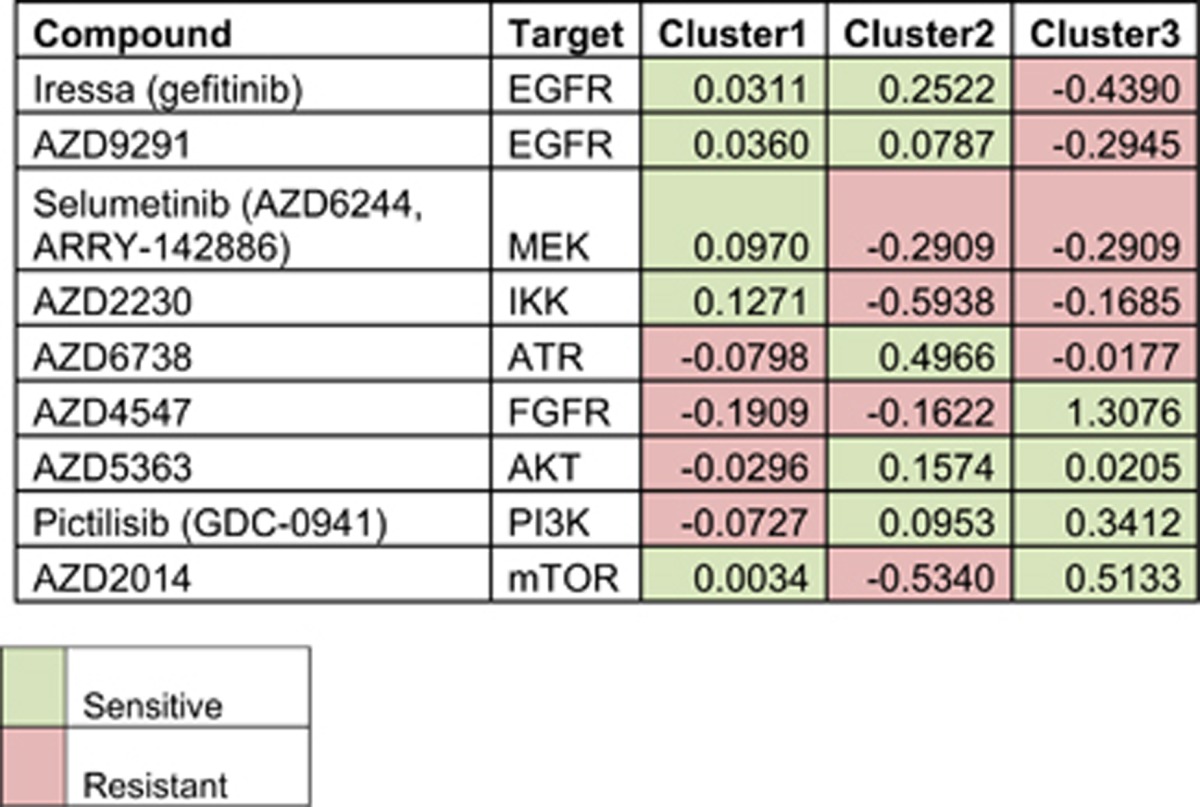
Compound sensitivity score

**Table 2:** Gene set enrichment analysis (GSEA)

*Gene set*	*TCGA* P*-value*	*Cell line* P*-value*	*Description*
*Cluster 1—Immune-evasion subtype*			
EGFR_UP.V1_UP	<0.001	<0.001	Genes upregulated in MCF-7 cells (breast cancer) positive for ESR1 and engineered to express ligand-activatable EGFR
RAF_UP.V1_UP	<0.001	<0.001	Genes upregulated in MCF-7 cells (breast cancer) positive for ESR1 MCF-7 cells (breast cancer) stably over-expressing constitutively active RAF1
HINATA_NFKB_IMMU_INF	<0.001	0.004	Immune or inflammatory genes induced by NF-kappaB in primary keratinocytes and fibroblasts
			
*Cluster 2—Replication-stress associated subtype*			
REACTOME_ACTIVATION_OF_ATR_IN_RESPONSE_TO_REPLICATION_STRESS	<0.001	0.005	Genes involved in activation of ATR in response to replication stress
NFE2L2.V2	0.093	<0.001	Genes upregulated in MEF cells (embryonic fibroblasts) with knockout of NFE2L2
SIRNA_EIF4GI_UP	0.432	<0.001	Genes upregulated in MCF10A cells vs knockdown of EIF4G1 gene by RNAi
			
*Cluster 3—Neuroendocrine subtype*			
CAHOY_NEURONAL	<0.001	0.036	Genes upregulated in neurons
BCAT_GDS748_DN	0.137	—	Genes regulated in HEK293 cells (kidney fibroblasts) expressing constitutively active form of CTNNB1 gene
BCAT_GDS748_UP	—	0.003	
ESC_V6.5_UP_EARLY.V1_UP	0.033	<0.001	Genes upregulated during early stages of differentiation of embryoid bodies from V6.5 embryonic stem cells

**Table 3:** microRNAs differentially expressed in both TCGA and cell lines

*Cell lines*			*TCGA*		
*MicroRNA*	*Fold change*	P*-value*	*MicroRNA*	*Fold change*	P*-value*
*Cluster 1—immune-evasion subtype*					
Upregulated miRs					
hsa-miR-30a	7.80	0.0211	hsa-miR-30a	1.64	1.58E−07
hsa-miR-29a	7.37	0.0144	hsa-miR-29a	1.44	5.45E−08
hsa-miR-29b	4.89	0.0166	hsa-miR-29b-2	1.36	0.0004605
		0.0166	hsa-miR-29b-1	1.38	0.0004026
hsa-miR-21	3.78	0.0049	hsa-miR-21	1.21	0.0000343
hsa-miR-29c	2.36	0.0175	hsa-miR-29c	1.36	0.0015875
hsa-miR-1976	1.10	0.0232	hsa-miR-1976	1.33	0.0018874
Downregulated miRs					
hsa-miR-1301	0.81	0.0065	hsa-miR-1301	0.58	1.62E−08
hsa-miR-345	0.76	0.0341	hsa-miR-345	0.60	1.52E−06
hsa-miR-454	0.72	0.0422	hsa-miR-454	0.78	0.0011665
hsa-miR-16	0.70	0.0389	hsa-miR-16-1	0.78	0.0022378
		0.0389	hsa-miR-16-2	0.69	3.48E−06
hsa-miR-421	0.65	0.0012	hsa-miR-421	0.61	9.57E−07
hsa-miR-944	0.60	0.0263	hsa-miR-944	0.42	0.0000337
hsa-miR-105	0.57	0.0058	hsa-miR-105-1	0.43	0.0053822
		0.0058	hsa-miR-105-2	0.40	0.0017061
hsa-miR-215	0.55	0.0132	hsa-miR-215	0.77	0.0362918
hsa-miR-192	0.52	0.0139	hsa-miR-192	0.58	4.74E−06
hsa-miR-194	0.50	0.0021	hsa-miR-194	0.71	0.0001613
hsa-miR-20a	0.49	0.0261	hsa-miR-20a	0.59	2.51E−09
hsa-miR-17	0.48	0.0195	hsa-miR-17	0.61	4.22E−11
hsa-miR-20b	0.46	0.0106	hsa-miR-20b	0.43	8.69E−07
hsa-miR-301b	0.45	0.0200	hsa-miR-301b	0.38	6.40E−12
hsa-miR-183	0.40	0.0278	hsa-miR-183	0.52	5.04E−11
hsa-miR-301a	0.36	0.0233	hsa-miR-301a	0.61	0.0000269
hsa-miR-363	0.30	0.0417	hsa-miR-363	0.65	0.0020219
hsa-miR-196b	0.20	0.0098	hsa-miR-196b	0.54	0.0001893
hsa-miR-9	0.16	0.0096	hsa-miR-9-2	0.42	4.51E−06
		0.0096	hsa-miR-9-1	0.42	0.0000041
hsa-miR-200a	0.11	0.0189	hsa-miR-200a	0.68	0.0000433
hsa-miR-429	0.09	0.0099	hsa-miR-429	0.62	0.0000735
hsa-miR-204	0.08	0.0222	hsa-miR-204	0.31	6.08E−07
hsa-miR-200b	0.08	0.0389	hsa-miR-200b	0.66	0.0000202
hsa-miR-200c	0.06	0.0403	hsa-miR-200c	0.77	0.000397
					
*Cluster 2—Replication-stress subtype*					
Upregulated miRs					
hsa-miR-205	221.50	0.0107	hsa-miR-205	3.35	5.56E−11
hsa-miR-203	14.99	0.0084	hsa-miR-203	2.77	5.08E−11
hsa-miR-944	3.03	0.0000	hsa-miR-944	5.15	5.37E−18
hsa-miR-149	3.02	0.0116	hsa-miR-149	2.34	2.92E−11
hsa-miR-663	2.53	0.0248	hsa-miR-663	1.41	0.0004309
hsa-miR-345	1.53	0.0073	hsa-miR-345	1.43	0.0005957
hsa-miR-3652	1.53	0.0057	hsa-miR-3652	1.31	0.0038245
hsa-miR-421	1.50	0.0389	hsa-miR-421	1.39	0.0008246
hsa-miR-513c	1.36	0.0173	hsa-miR-513c	1.57	0.0000372
hsa-miR-3687	1.33	0.0389	hsa-miR-3687	1.60	0.0001114
hsa-miR-378b	1.14	0.0370	hsa-miR-378b	1.28	0.0008767
Downregulated miRs					
hsa-miR-181b	0.24	0.0389	hsa-miR-181b	0.68	5.2E−08
hsa-miR-181a	0.18	0.0161	hsa-miR-181a	0.71	2.33E−07
					
*Cluster 3—Neuroendocrine subtype*					
Upregulated miRs					
hsa-miR-9	15.51	0.0014	hsa-miR-9-2	3.55	4.31E−08
		0.0014	hsa-miR-9-1	3.53	4.47E−08
hsa-miR-335	6.67	0.0109	hsa-miR-335	1.62	0.0000279
hsa-miR-19b	5.70	0.0012	hsa-miR-19b-1	1.34	0.0136969
		0.0012	hsa-miR-19b-2	1.33	0.004951
hsa-miR-301b	4.50	0.0000	hsa-miR-301b	1.75	0.0019509
hsa-miR-17	3.78	0.0002	hsa-miR-17	1.46	0.0000667
hsa-miR-20a	3.67	0.0004	hsa-miR-20a	1.48	0.0004789
hsa-miR-18a	3.50	0.0164	hsa-miR-18a	1.67	0.0000272
hsa-miR-20b	3.49	0.0005	hsa-miR-20b	1.89	0.0031859
hsa-miR-18b	2.89	0.0060	hsa-miR-18b	1.47	0.0030779
hsa-miR-592	2.74	0.0024	hsa-miR-592	1.48	0.0266718
hsa-miR-92a	2.47	0.0063	hsa-miR-92a-2	1.63	0.0000072
		0.0063	hsa-miR-92a-1	1.73	0.0000134
hsa-miR-105	2.38	0.0003	hsa-miR-105-2	4.31	0.0000456
		0.0003	hsa-miR-105-1	5.36	0.0000057
hsa-miR-194	2.13	0.0174	hsa-miR-194-2	1.38	0.0044099
		0.0174	hsa-miR-194-1	1.36	0.00697
hsa-miR-181c	1.74	0.0311	hsa-miR-181c	1.44	0.0000283
hsa-miR-1301	1.29	0.0138	hsa-miR-1301	1.38	0.0088744
Downregulated miRs					
hsa-miR-424	0.23	0.0198	hsa-miR-424	0.80	0.040225
hsa-miR-222	0.18	0.0023	hsa-miR-222	0.64	7.04E−06
hsa-miR-221	0.18	0.0016	hsa-miR-221	0.68	0.0000452
hsa-miR-23a	0.14	0.0001	hsa-miR-23a	0.75	6.79E−06
hsa-miR-27a	0.13	0.0002	hsa-miR-27a	0.70	0.0000629
hsa-miR-21	0.10	5.27E-07	hsa-miR-21	0.73	1.12E−08
hsa-miR-22	0.08	0.000038	hsa-miR-22	0.71	7.96E−08
hsa-miR-29b	0.05	0.0000397	hsa-miR-29b-2	0.68	0.0002611
		0.0000397	hsa-miR-29b-1	0.66	0.0001657
hsa-miR-29a	0.02	0.0000187	hsa-miR-29a	0.74	0.0003523
