# Supplementary material for: Enhanced MAPK signaling drives ETS1-mediated induction of miR-29b leading to downregulation of TET1 and changes in epigenetic modifications in a subset of lung SCC
Source: Oncogene. 2016 Jan 18;35(33):4345–57. doi: 10.1038/onc.2015.499 (PMC4994018; doi:10.1038/onc.2015.499)
Supplement: Supplementary Figure S1 [file onc2015499x1.pdf]

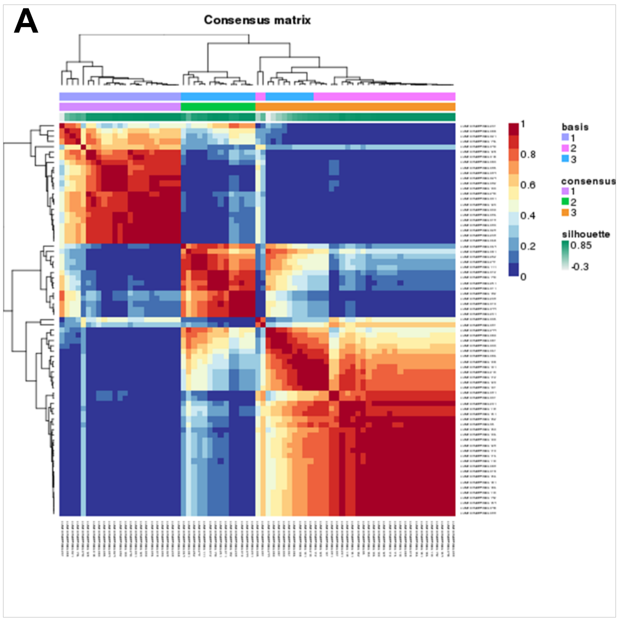

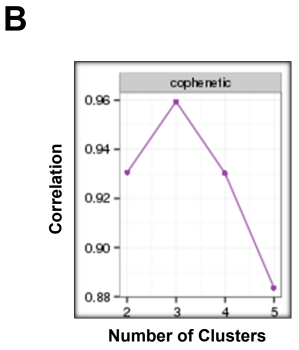

C

Gene Set Enrichment Analysis (GSEA)

NAME	SIZE	ES	NES	NOM p-val	FDR q-val	FWER p-val	RANK AT MAX
**Cluster 1 - Immune-evasion subtype**							
EGFR_UP.V1_UP	128	0.528049	1.410282	0.059867	0.080034	0.695	2236
RAF_UP.V1_UP	129	0.538945	1.530657	0.016563	0.101432	0.365	1628
MANTOVANI_NFKB_TARGETS_UP	34	0.636125	1.549249	0.024641	0.189177	0.994	1607
**Cluster 2 - Replication-stress associated subtype**							
REACTOME_ACTIVATION_OF_ATR_IN_RESPONSE_TO_REPLICATION_STRESS	26	0.450506	0.888432	0.599589	0.940041	1	2154
NFE2L2.V2	274	0.468384	1.477759	0.029907	0.461163	0.535	1520
SIRNA_EIF4GI_UP	48	0.223866	0.715286	0.893788	0.964822	1	2749
**Cluster 3 - Neuroendocrine subtype**							
GCNP_SHH_UP_LATE.V1_UP	119	0.416491	1.373997	0.055777	0.275706	0.767	1658
BCAT_BILD_ET_AL_UP	30	0.338917	0.867011	0.654649	0.781816	1	2310

Supplementary Figure S1: (A) iNMF on SCC samples extracted from the Lee et. al. dataset (GSE8894) indicates that there are three clusters (subtypes) found in an independent dataset. (B) Cophenetic plot indicates that three clusters is the optimal solution for this dataset. (C) GSEA results indicating similar pathway enrichment profiles to the TCGA dataset.
