# Supplementary material for: Enhanced MAPK signaling drives ETS1-mediated induction of miR-29b leading to downregulation of TET1 and changes in epigenetic modifications in a subset of lung SCC
Source: Oncogene. 2016 Jan 18;35(33):4345–57. doi: 10.1038/onc.2015.499 (PMC4994018; doi:10.1038/onc.2015.499)
Supplement: Supplementary Figure S2 [file onc2015499x2.pdf]

A

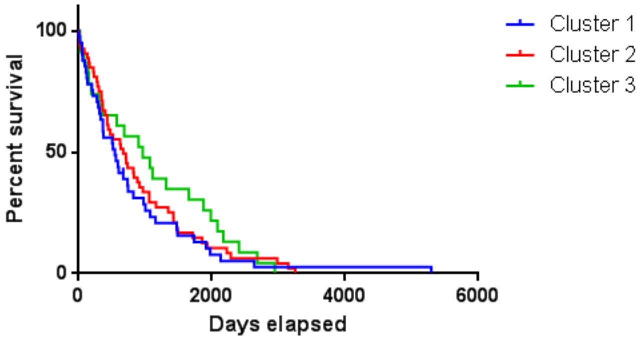

B

Cluster	stage i	stage ia	stage ib	stage iia	stage iib	stage iiia	stage iiib	stage iv
1	0.00%	17.02%	37.23%	7.45%	15.96%	13.83%	6.38%	2.13%
2	0.92%	11.01%	41.28%	6.42%	20.18%	11.93%	7.34%	0.92%
3	0.00%	21.57%	33.33%	11.76%	15.69%	9.80%	5.88%	1.96%

C

Cluster	Early (Stage I and II)	Late (Stage III and IV)
1	77.66%	22.34%
2	79.82%	20.18%
3	82.35%	17.65%

D

Cluster	Average number of pack years smoked
1	54.90
2	48.47
3	62.71

Supplementary Figure S2: SCC clusters patient samples do not show a difference in survival, smoking status, or stage. (A) Kaplan-Meier plots of overall survival from patients belonging to each cluster. (B) Percent of patients with each stage of disease in each cluster. (C) Percent of patients with early vs. Late stage of disease in each cluster. (D) Average number of pack years smoked from patients from each cluster. All data was taken from the available patient meta-data from the TCGA.
