# Supplementary material for: Enhanced MAPK signaling drives ETS1-mediated induction of miR-29b leading to downregulation of TET1 and changes in epigenetic modifications in a subset of lung SCC
Source: Oncogene. 2016 Jan 18;35(33):4345–57. doi: 10.1038/onc.2015.499 (PMC4994018; doi:10.1038/onc.2015.499)
Supplement: Supplementary Figure S3 [file onc2015499x3.pdf]

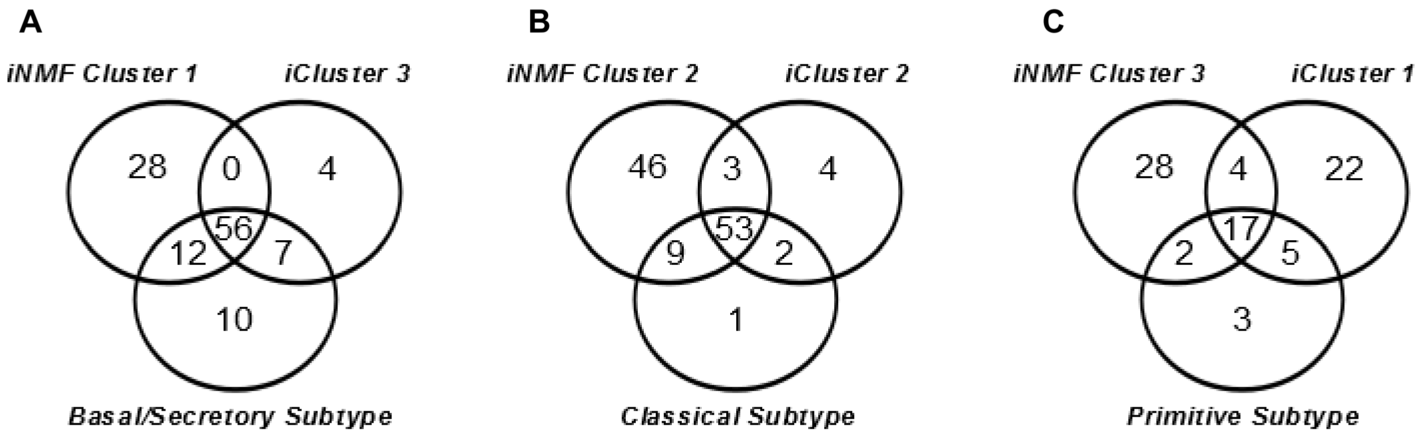

Supplementary Figure S3: Overlap of TCGA samples by different clustering methods. (A) Venn diagram depicting the number of TCGA patient samples that overlap with iNMF Cluster 1 using iClustering from the TCGA and the subtyping described by Wilkerson *et. al.* **.** (B) Venn diagram depicting the number of TCGA patient samples that overlap with iNMF Cluster 2 using iClustering from the TCGA and the subtyping described by Wilkerson *et. al.* (C) Venn diagram depicting the number of TCGA patient samples that overlap with iNMF Cluster 2 using iClustering from the TCGA and the subtyping described by Wilkerson *et. al.*
