# Supplementary material for: Enhanced MAPK signaling drives ETS1-mediated induction of miR-29b leading to downregulation of TET1 and changes in epigenetic modifications in a subset of lung SCC
Source: Oncogene. 2016 Jan 18;35(33):4345–57. doi: 10.1038/onc.2015.499 (PMC4994018; doi:10.1038/onc.2015.499)
Supplement: Supplementary Figure S4 [file onc2015499x4.pdf]

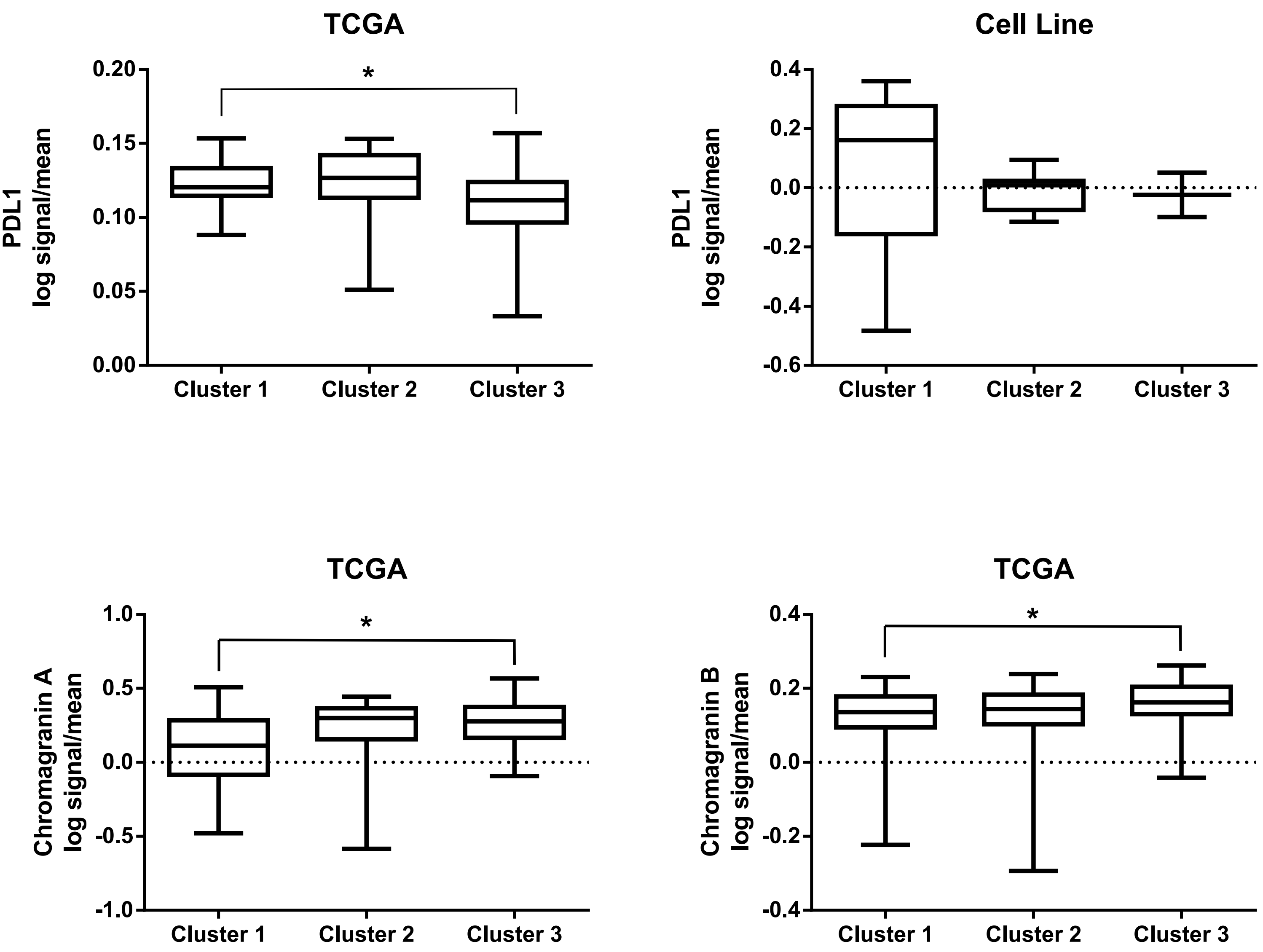

Supplementary Figure S4: PD-L1 and Chromagranin expression across the clusters. (A) PD-L1 expression in TCGA samples groupled by cluster (*,p<0.05 by Student's t-test). (B) PD-L1 expression in cell line samples groupled by cluster. (C) Chromagranin A expression in TCGA samples groupled by cluster (*,p<0.05 by Student's t-test). (D) Chromagranin B expression in TCGA samples groupled by cluster (*,p<0.05 by Student's t-test).
