# Supplementary material for: Enhanced MAPK signaling drives ETS1-mediated induction of miR-29b leading to downregulation of TET1 and changes in epigenetic modifications in a subset of lung SCC
Source: Oncogene. 2016 Jan 18;35(33):4345–57. doi: 10.1038/onc.2015.499 (PMC4994018; doi:10.1038/onc.2015.499)
Supplement: Supplementary Figure S5 [file onc2015499x5.pdf]

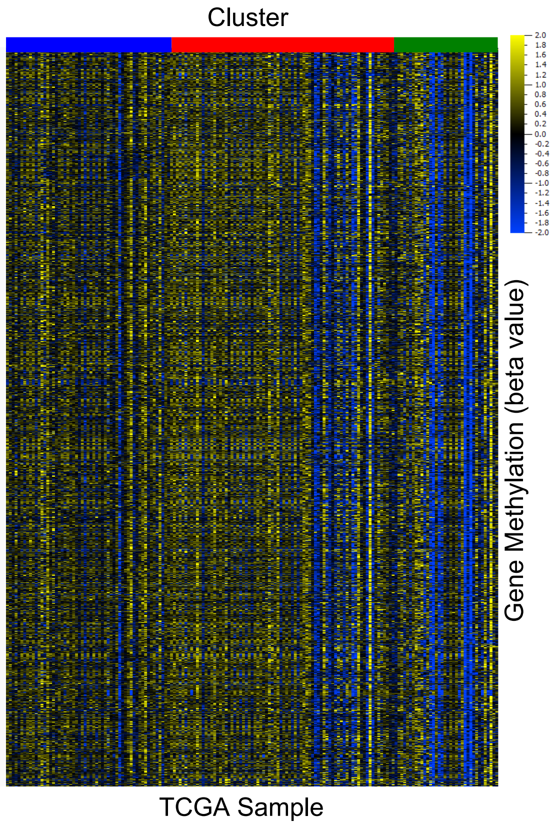

Supplementary Figure S5: Methylation by mean beta values were downloaded from The TCGA and iNMF cluster genes were extracted for further analysis. iNMF genes that were differentially methylated between the three clusters (ANOVA $p<0.05$) are plotted and the data values are available in Supplementary Table S6.
