# Supplementary material for: Enhanced MAPK signaling drives ETS1-mediated induction of miR-29b leading to downregulation of TET1 and changes in epigenetic modifications in a subset of lung SCC
Source: Oncogene. 2016 Jan 18;35(33):4345–57. doi: 10.1038/onc.2015.499 (PMC4994018; doi:10.1038/onc.2015.499)
Supplement: Supplementary Figure S6 [file onc2015499x6.pdf]

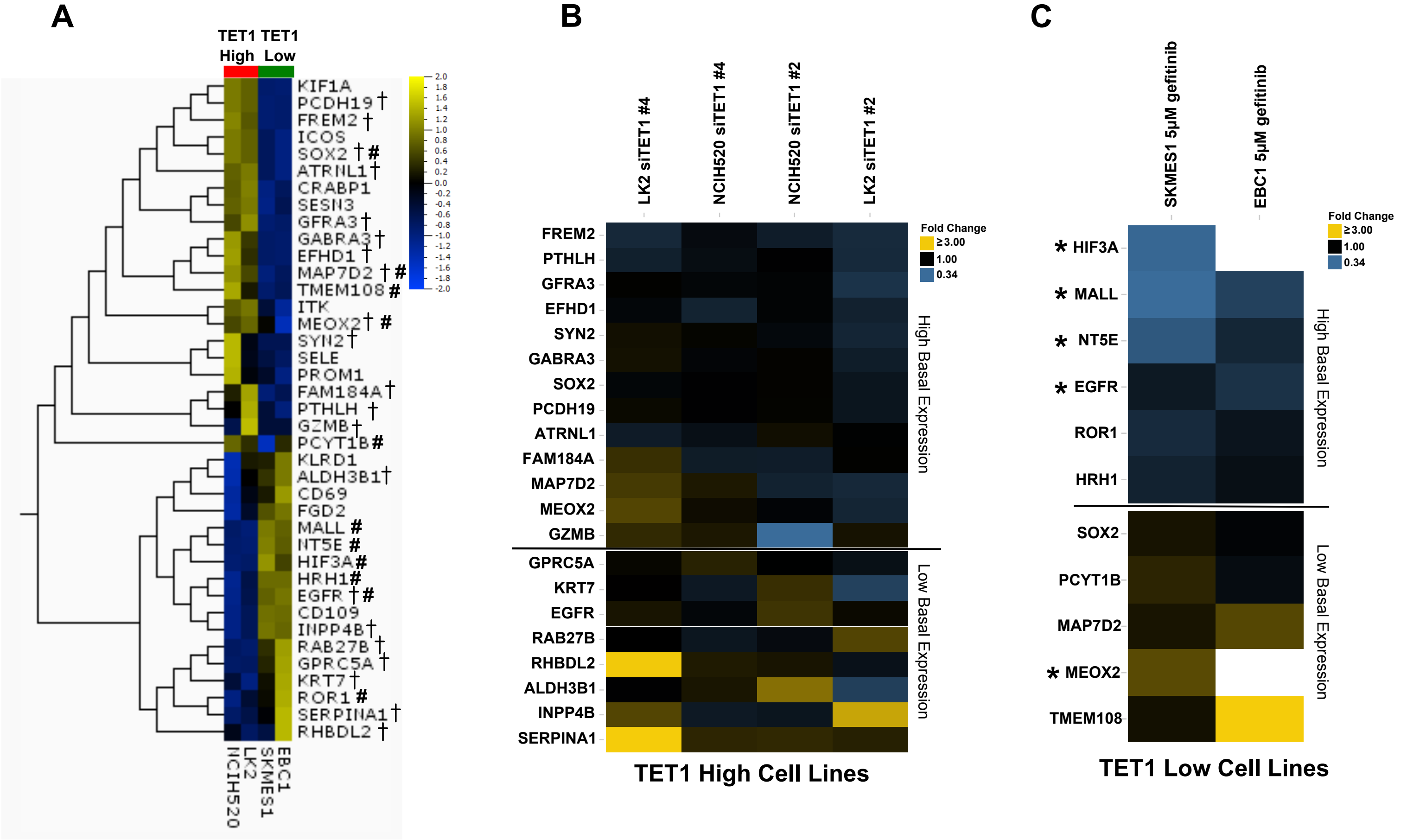

Supplementary Figure S6: Gene expression changes after TET1 modulation. (A) A subset of the iNMF signature genes, that also showed modulation by TET1 knockdown in at least one previous study (GSE24843, GSE26830, and GSE50016) and were found to be significantly differentially expressed between immune-evasion (EBC1 and SKMES1) and neuroendocrine (LK2 and NCIH520) subtype cell lines were chosen for further analysis. **(B)** LK2 and NCIH520 cells were collected 72 hours after siRNA knockdown of TET1 or siRNA control and changes in expression of the genes defined in (A) were measured by fluidigm chip PCR. All data is the mean of n=3 where expression change was normalized to siControl. **(C)** EBC1 and SKMES1 cells were treated with gefitinib for 72 hours (increasing TET1 expression) after which point gene expression changes in the genes defined in (A) were measured by fluidigm chip PCR. All data is the mean of n=3 where expression change was normalized to vehicle treated cells. †=genes changed by TET1 knockdown, # = genes changed by gefitinib treatment, *$p<0.05$ based on a 2 way students t-test by TET1 siRNA compared to siControl.
