# Supplementary material for: Enhanced MAPK signaling drives ETS1-mediated induction of miR-29b leading to downregulation of TET1 and changes in epigenetic modifications in a subset of lung SCC
Source: Oncogene. 2016 Jan 18;35(33):4345–57. doi: 10.1038/onc.2015.499 (PMC4994018; doi:10.1038/onc.2015.499)
Supplement: Supplementary Figure S7 [file onc2015499x7.pdf]

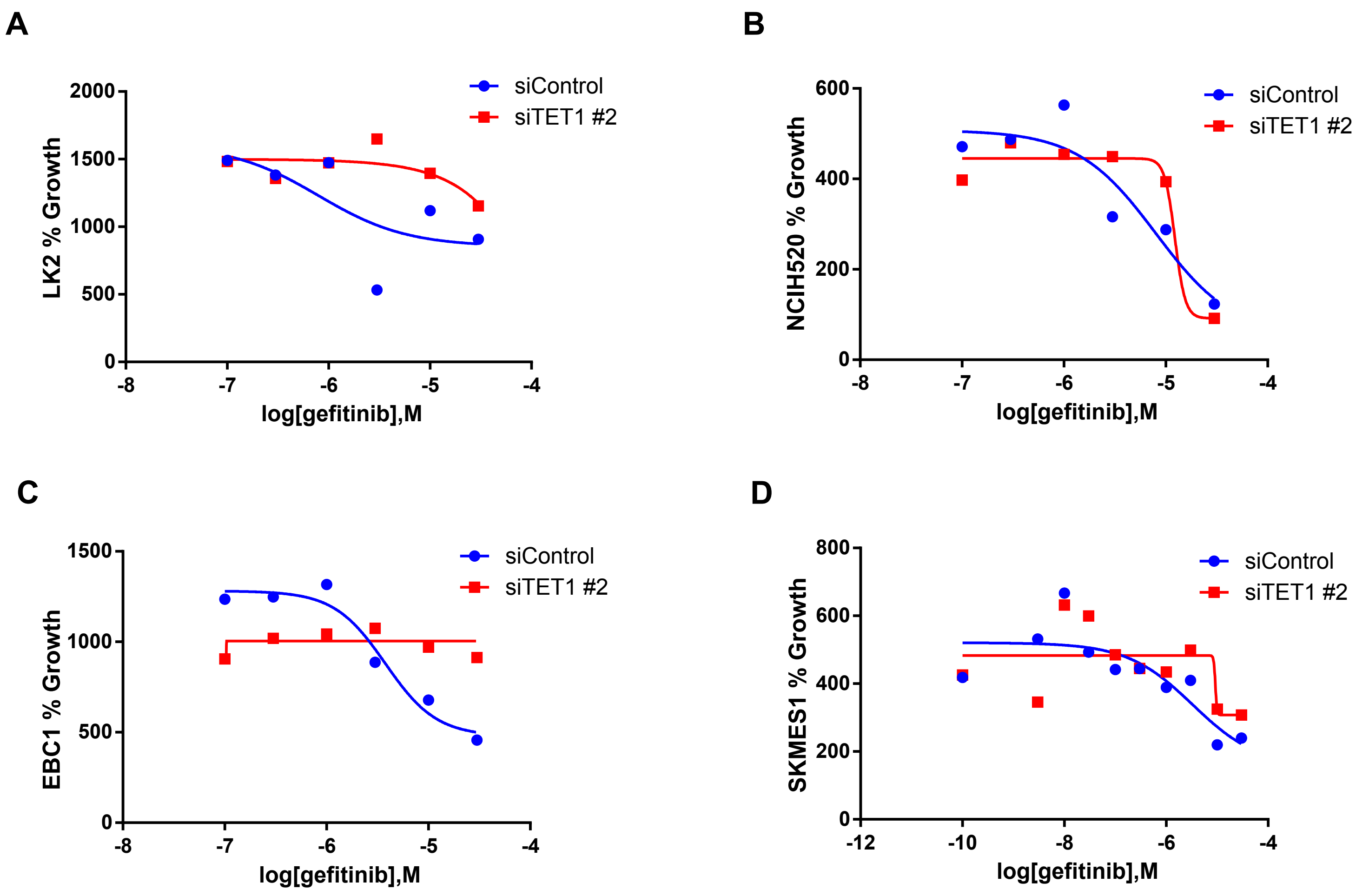

Supplementary Figure S7: Response to gefitinib after TET1 knockdown. **(A)** LK2, **(B)** NCIH520, **(C)** EBC1, and **(D)** SKMES1 cells were transfected with siRNA aganst TET1 or Control siRNA and treated with indicated concentrations of gefitinib the following day. Confluence was measured over a period of 120 hours using the incucyte. Proliferation was measured as a percent of confluence normalized to the confluence at 0 hours. All data is the mean of n=4.
