# Supplementary material for: Enhanced MAPK signaling drives ETS1-mediated induction of miR-29b leading to downregulation of TET1 and changes in epigenetic modifications in a subset of lung SCC
Source: Oncogene. 2016 Jan 18;35(33):4345–57. doi: 10.1038/onc.2015.499 (PMC4994018; doi:10.1038/onc.2015.499)
Supplement: Supplementary Table Legends [file onc2015499x20.doc]

**Supplementary Table S1: iNMF cluster defining genes found in the TCGA dataset**. Column 1 indicates genes found by iNMF to define the clusters specified in column 2.

**Supplementary Table S2: iNMF cluster defining genes in the GSE8894 validation data set.**  Column 1 indicates genes found by iNMF to define the clusters specified in column 2.

**Supplementary Table S3: Cell Line IC50 Values.** Indicates the individual IC50 values and the cluster IC50 values used to calculate the compound sensitivity score shown in Table 1.

**Supplementary Table S4: Gene Set Enrichment Analysis (GSEA) results.** Gene set enrichment scores for TCGA and cell lines are indicated.

**Supplementary Table S5: Differential microRNA expression values.**  This table indicates microRNAs that were differentially expressed between the three subtypes in TCGA and cell lines as defined by an ANOVA test with a cutoff of p<0.05. p-values and q-values for each microRNA are shown.

**Supplementary Table S6: Transcription Factor Master List.** T-tests were performed on expression of transcription factors predicted to bind to microRNA promoters. Transcription factors that showed statistically significant p<0.05 expression between two clusters are highlighted in green. The clusters that are being compared are indicated by the “Level” and “-Level” columns.

**Supplementary Table S7: microRNA expression after ETS1 knockdown.**  R-squared values for correlation between ETS1 and each microRNA are shown for both TCGA and cell lines are indicated. In addition the results of t-tests performed on fold change expression data after ETS1 knockdown for the data presented in Figure 4 are shown. Statistically significant results (p<0.05) are highlighted in green.

**Supplementary Table S8: Differentially methylated genes.** List of genes identified by iNMF to be differentially expressed between clusters that are also differentially methylated between clusters. The gene name, the cluster, and the p-values are indicated.

**Supplementary Table S9: Gene expression changes after TET1 knockdown.** The difference, standard error, and t-test results for mRNA changes after TET1 knockdown in LK2 and NCIH520 cells are shown by gene name.

**Supplementary Table S10: Gene expression changes after gefitinib treatment.** The difference, standard error, and t-test results for mRNA changes after EBC1 and SKMES1 cell lines were treated with gefitinib are shown. Significant (p<0.05) changes are highlighted in green.

**Supplementary Table S11: Primers and Probes.** Listed are the primers and probes used for expression analysis.

**Supplementary Table S12: Antibodies.** Listed are the antibodies used for western blots.
